# Dataset supporting blood pressure prediction for the management of chronic hemodialysis

**DOI:** 10.1038/s41597-019-0319-8

**Published:** 2019-12-09

**Authors:** Cheng-Jui Lin, Ying-Ying Chen, Chi-Feng Pan, Vincent Wu, Chih-Jen Wu

**Affiliations:** 1 Division of Nephrology, Department of Internal Medicine, MacKay Memorial Hospital, Taipei, Taiwan; 2 Department of Medicine, Mackay Medical College, New Taipei, Taiwan; 3 Mackay Junior College of Medicine, Nursing and Management, Taipei, Taiwan; 4 Division of Nephrology, Department of Internal Medicine, National Taiwan University Hospital, Taipei, Taiwan; 5 Department of Medical Research, China Medical University Hospital, China Medical University, Taichung, Taiwan

**Keywords:** End-stage renal disease, Haemodialysis

## Abstract

Hemodialysis (HD) is a treatment given to patients with renal failure. Notable treatment-related complications include hypotension, cramps, insufficient blood flow, and arrhythmia. Most complications are associated with unstable blood pressure during HD. Physicians are devoted to seeking solutions to prevent or lower the incidence of possible complications. With advances in technology, big data have been obtained in various medical fields. The accumulated dialysis records in each HD session can be gathered to obtain big HD data with the potential to assist HD staff in increasing patient wellbeing. We generated a large stream of HD parameters collected from dialysis equipment associated with the Vital Info Portal gateway and correlated with the demographic data stored in the hospital information system from each HD session. We expect that the application of HD big data will greatly assist HD staff in treating intradialytic hypotension, setting optimal dialysate parameters, and even developing an intelligent early-warning system as well as providing individualized suggestions regarding dialysis settings in the future.

## Abstract (summary)

**Table:** 

Measurement(s)	blood pressure • bioinformatics analysis
Technology Type(s)	dialysis system • computational modeling technique
Factor Type(s)	sex • age
Sample Characteristic - Organism	Homo sapiens

Machine-accessible metadata file describing the reported data: 10.6084/m9.figshare.10311188

## Background & Summary

The global population with end-stage renal disease (ESRD) is increasing annually. Hemodialysis, which provides the excellent, rapid clearance of solutes, is the most commonly used renal replacement therapy for those who need dialysis. Blood pressure (BP) variation is frequently encountered and is associated with most complications during regular hemodialysis (HD)^1^. Both intradialysis hypotension and hypertension have strong implications for adverse outcomes, including increased cardiovascular events and overall morbidity and mortality^2–5^. A few observational studies have confirmed the “U-shaped” or “reverse J-shaped” relationship between BP and mortality in a variety of ESRD cohorts^6–8^.

However, dialysis-associated hypotension is considered to be a frequent complication ranging from 5.6 to 76.7% at each HD session and may contribute to considerable symptom burden^9^.One report has shown that 75% of HD patients had at least one episode of hypotension^10^. Another study revealed that more than 50% of treatments were complicated by intradialytic hypotension^11^. Intradialytic hypotension is associated with older patients with diabetes and lower predialysis BP and longer dialysis vintage^3,10^. Although the exact mechanisms are poorly understood, the possible mechanisms include an increased left ventricular mass index, extracellular volume overload, sympathetic overactivity, sodium loading from dialysate, and the use of antihypertensive medications during HD^12–17^.

The decrease in blood volume occurring during the initial dialysis process can result in hypotension. The Kidney Disease Outcomes Quality Initiative (KDOQI) guidelines define intradialytic hypotension as a decline in systolic BP (SBP) > 20 mmHg or a decrease in the mean arterial BP > 10 mmHg associated with symptoms^18^. However, because symptoms and intervention data are often unavailable in large databases, some intradialytic hypotension definitions are exclusively based on SBP measurements^19^. Patients with hypotension might experience muscle cramps, nausea, vomiting, yawning, sighing, lightheadedness and hoarseness before the decline in BP^18^. These symptoms are easily overlooked during the regular dialysis process. Individuals with severe hypotension might develop vascular access thrombosis and experience inadequate dialysis or even bowel ischemia but not cardiovascular events^20,21^.

A number of strategies have been suggested to prevent the risk of intradialytic hypotension^11^. These strategies include minimizing interdialytic weight gain, discontinuing antihypertensive medications prior to dialysis, and not eating during dialysis^22^. These methods, however, did not markedly reduce the incidence of interdialytic hypotension^9^. Thus, avoiding and/or lowering the frequency of this serious complication is an essential issue for physicians and nurses in dialysis centers. With the advent of the era of big data and artificial intelligence, there are few reports applying the concept of HD big data in HD. We aimed to show the process and method of collecting data, including vital signs, dialysis settings, and demographic data, during HD treatment. We further aimed to clean and analyze the dataset. Due to the massive volume and complexity of the data, the Wistron Corporation Bestshape^®^ Dialysis Assistant System, comprising two parts, the Vital Info Portal (VIP) gateway device and a medical informatics analysis system program, was used.

This study is the largest to date to introduce the concept of HD big data in the field of hemodialysis. In the future, this valuable data set may be used to develop an intelligent early-warning system capable of predicting BP changes, setting optimal dialysate parameters, and finally providing timely and individualized suggestions regarding dialysis settings.

## Methods

We recruited outpatient subjects from the HD unit of MacKay Memorial Hospital, a tertiary medical center in Taiwan. The study period was from 2013/06 to 2018/07. The project and experimental procedures were approved by the institutional review board of our hospital (16MMHIS044). The search followed the entire compliance guidelines for good clinical practice and complied with the ethical principles of the Declaration of Helsinki. Clinical data were collected after a regular dialysis session. Informed consent was obtained from all study patients. We excluded subjects under 20 years old since adult patients were more appropriate for a chronic disease study. Supplementary information on patient HD risk factors, clinical symptoms, and previous medical history as well as all relevant records and any correlated data were gathered in this study. All data were de-identified to protect patient secrecy in this retrospective study. Data from 1,075 outpatients who underwent a total of 4,366,298 HD recordings in 165,986 HD sessions were collected between 2013/06 and 2018/07.

The default dialysate sodium concentration was 138 mmol/L, the dialysate calcium concentration was 3.0 mmol/L, the dialysate flow rate was 500 ml/min, and the dialysate temperature was 36.5 °C for most patients. Bicarbonate-containing dialysate and biocompatible artificial kidneys were used for all patients. During HD, BP, pulse rate, and body temperature were measured. BP and pulse rate were taken several times by an electronic sphygmomanometer from the session start to the end at 30-minute intervals and additionally thereafter according to clinical need, e.g., in the case of cramps. Body temperature was checked using an ear thermometer. The vital signs were measured at each time point, and concurrent dialysis settings, including blood flow rate, ultrafiltration rate, total ultrafiltration volume, dialysate temperature, and dialysate sodium concentration, were recorded. If the measured SBP was out of range (SBP was greater than 200 or less than 30), then the SBP was measured again, and the latest reading was recorded.

**Fig. 1:**
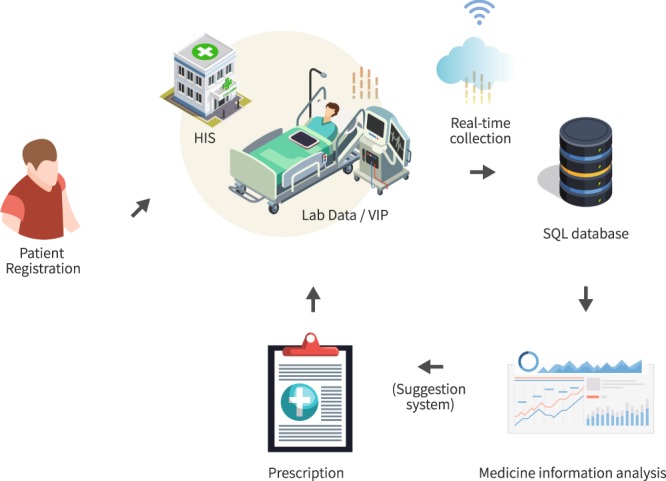
Schematic flow chart of the big HD data generation. The data were collected from dialysis equipment connected to the Vital Info Portal (VIP) gateway and linked with demographic data stored in the hospital information system for each HD session. Finally, the results analyzed from this dataset can be applied to assist the HD staff in treating clinical complications and setting up individualized suggestions regarding dialysis settings in the future.

**Fig. 2:**
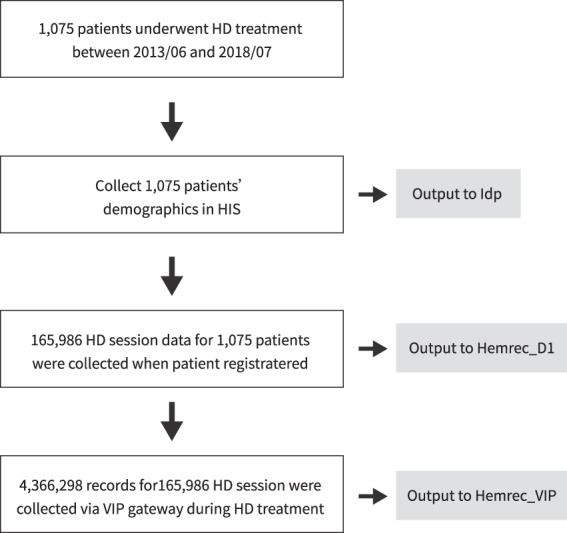
Flow chart of the HEMOBP data collection process.

Figures 1 and 2 outline the data collection process and data collection flow. The subjects underwent HD treatments two to three times a week, with each treatment lasting up to 240 minutes. All patients met the inclusion criteria, and all relevant data were obtained.

## Data Records

**Fig. 3:**
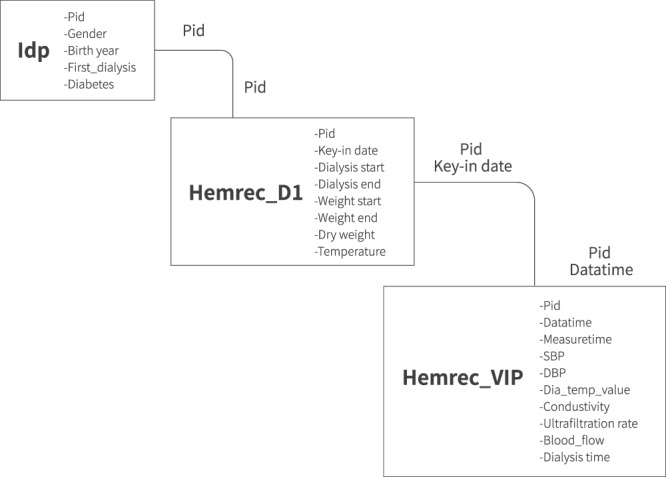
Entity relationship diagram of the HEMOBP dataset.

The data sources were recorded in three tables, Hemrec_D1, Hemrec_VIP, and Idp and are available at figshare^23^. Idp, which records the patient demographics, was from HIS. The patient health status for an HD session, including their weight, dry weight, body temperature, etc., was collected from a dialysis assistant system. Furthermore, Hemrec_VIP collected data from the HD machine directly via the VIP gateway. The relationships of the three tables are shown in Fig. 3. The Pid was the unique id used to link the patient information in the Idp and Hemrec_D1 tables. The Pid and HD data were used to connect the Hemrec_D1 and Hemrec_VIP tables. The values were input into a spreadsheet containing various fields:

1. Idp describes patient demographics, including the following:
  Pid: Unidentified patient ID.
  Gender: Patient gender.
  Birth year: Patient birth year.
  First_dialysis: The date the patient underwent HD treatment for the first time. Diabetes: Patient with/without diabetes.
  The key to linking Idp and Hemrec_D1 was the “Pid”; the key to merging Hemrec_D1 and Hemrec_VIP was the Pid and the date (key-in date in Hemrec_D1 and data time in Hemrec_VIP).
2. The Hemrec_D1 table records the patient health status
  The columns are listed and described as follows:
  Pid: Unidentified patient ID.
  Key-in date: Date of the data record.
  Dialysis start: Start time of each HD session.
  Dialysis end: End time of each HD session.
  Weight start: Body weight before each HD session.
  Weight end: Body weight after each HD session.
  Dry weight: Goal body weight without fluid overload or hypovolemia. Temperature: Body temperature.
3. The Hemrec_VIP collected data from the VIP gateway

The columns were as follows:

Pid: Unidentified patient ID.

Data time: Date of the data record.

Measure time: Times for measuring BP.

SBP: Patient systolic blood pressure.

DBP: Patient diastolic blood pressure.

Dia_temp_value: Dialysate setting temperature.

Conductivity: A parameter for the sodium concentration in the dialysate.

Ultrafiltration rate: The rate of ultrafiltration (ultrafiltration-fluid removal during hemodialysis).

Blood_flow: The rate of blood flow through dialyzers and associated devices. Dialysis time: Elapsed time of HD: The duration from start to end of each HD session.

## Technical Validation

Due to the massive volume and complexity of the data, we utilized the Wistron Corporation Bestshape^®^ Dialysis Assistant System, comprising two parts, the VIP gateway device and a medical informatics analysis system program. The VIP gateway is a device that collects vital signs and corresponding dialysis machine settings. Then, the VIP uploads the records to a data lake located in the dialysis center. All data were double-checked on-site by a team of two nurses and were gathered electronically via the VIP gateway. Structured Query Language (SQL) was used to manage individual electronic medical records, which were then stored in the Oracle database.

Other clinical information, including age, gender, and diabetes mellitus, was also collected in the Hospital Information System (HIS). The data collection process was similar to a conventional SBP clinical study, but the analysis and results were completely different. A conventional study was performed in the time domain, whereas this system was processed in the time-frequency domain. That is, the dataset was not collected in a fixed time interval in different HD sessions and patients. When a patient experienced some symptoms in which the clinic nurse had to change the dialysate setting and then measure the BP, the new machine setting or BP records were recorded in the respective dataset. Hence, we suggest that the use of HEMOBP should consider the time-frequency domain. Although the SBP was processed in the time-frequency domain, the actual condition of the underlying SBP can be detected with high accuracy. This implementation allowed us to build in the R statistical programming environment and conduct visualization using the Shiny web application. We developed an interactive and dynamic web application for a differential HD treatment session.

With the ongoing advancements in artificial intelligence and machine-learning techniques, our data can be applied to different HD issues. This application enabled model selection, model prediction, parameter tuning, and the visualization of the results in a user-friendly interface based on this dataset. The construction of an SBP prediction intelligent system through this HD dataset could aid in the clinical decision-making process and further help reduce the incidence of intradialytic hypotension^23^. The future application of these models may facilitate SBP-targeted outcome studies and shed light on the reduction in the frequency of intradialytic SBP variability and associated comorbidities.

**Fig. 4:**
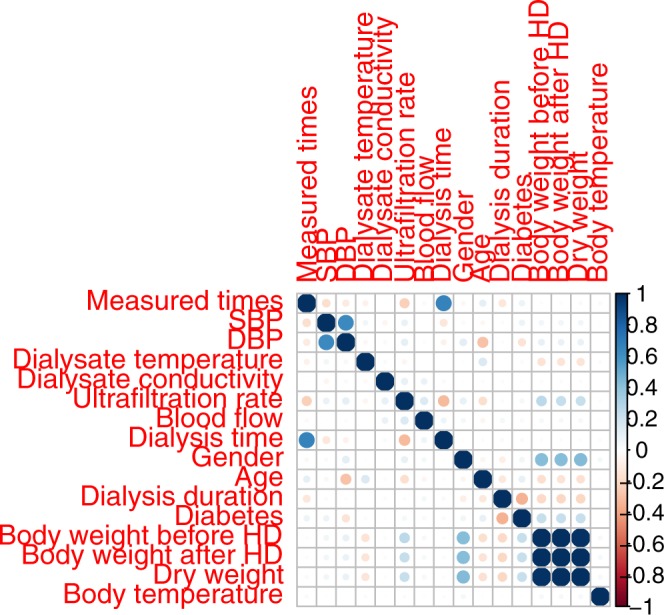
The plot with mutual correlation compared among independent parameters collected in the HEMOBP dataset.

**Fig. 5:**
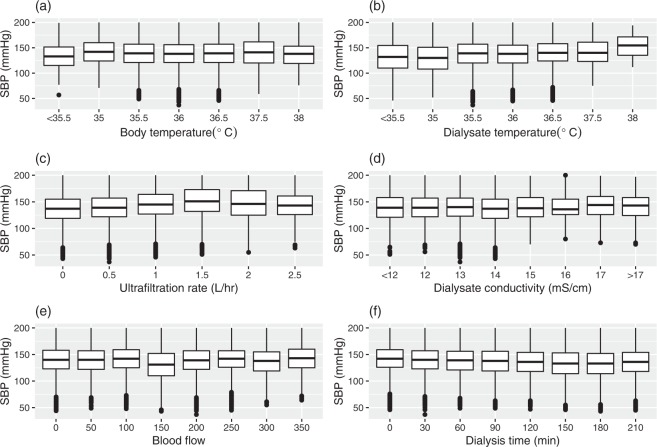
The correlation between SBP and independent HD parameters during HD treatment. (**a**) Graph drawn by SBP and body temperature. (**b**) Graph drawn by SBP and dialysate temperature. **(c**) Graph drawn by SBP and ultrafiltration rate. (**d**) Graph drawn by SBP and dialysate conductivity. (**e)** Graph drawn by SBP and blood flow. (**f**) Graph drawn by SBP and dialysis time.

The primary research endpoint was to present the collection of retrospective clinical HD big data. As all cases collected included clinical characteristics, these data can be further applied in many ways, including machine learning and the featured extraction process to find patterns correlated specifically with SBP detection. Statistical analysis was provided for all parties as the means ± standard deviations for continuous data and number (percentage) for categorical data. The correlations between clinical variables were represented by the correlation plot of the SBP dataset. Table 1 shows the data distribution in the three data sets. The averages for the SBP and DBP were 137.68 ± 25.85 and 68.33 ± 14.33 mmHg, respectively. All demographic distributions are listed in Table 1. Laboratory biochemistry test results were not included since these tests were performed only once a month, whereas for the BP records, we collected data from each HD session. Figure 4 represents the correlation plot between independent parameters collected in the HEMOdialysis Blood Pressure (HEMOBP) dataset. Figure 5 shows the association between SBP and other independent HD parameters, including body temperature, dialysate temperature, ultrafiltration rate, dialysate conductivity, blood flow and dialysis time during HD treatment.

**Table 1:** Characteristics of the patients included in the HEMOBP dataset.

Number of HD patients	1,075
Number of BP records	4,366,298
Male	527 (49.16%)
Diabetes mellitus (%)	370 (34.51%)
Measured times (hours)	4.66 ± 3.66
Dry weight (kg)*	58.4 ± 13.55
Body temperature (°C)	36.4 ± 0.27
Body weight before HD (kg)	61.07 ± 14.39
Body weight after HD (kg)	58.75 ± 13.87
Ultrafiltration rate (L/h)	0.52 ± 0.41
Blood flow (ml/min)	186.82 ± 98.04
Dialysate temperature (°C)	36.39 ± 0.45
Dialysate conductivity (mS/cm)***	14.02 ± 0.38
Systolic blood pressure (mmHg)	137.68 ± 25.85 Diastolic
blood pressure (mmHg)	68.33 ± 14.33

Values are expressed as the means ± standard deviations (SD) for continuous data. Dry weight *: goal of body weight without fluid overload or hypovolemia; Dialysate conductivity ***: a parameter of sodium concentration in dialysate.

## Usage Note

**Fig. 6:**
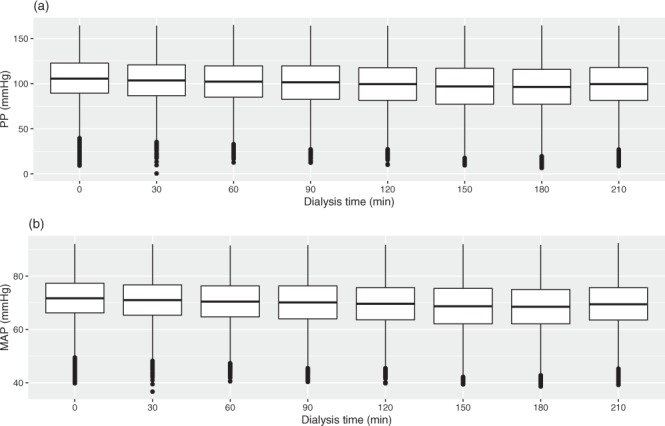
The association between blood pressure and dialysis time with a 30-minute interval during one HD session. (**a**) Graph drawn by PP and dialysis time. (**b**) Graph drawn by MAP and dialysis time.

We provide a longitudinal dataset of HD treatments, including demographic and real-time dialysis settings. As in previous reports on HD big data, the following BP change will be predicted based on the current setting and the patient health information^24^. Users should note that the variables could be computed by their study of interest. For example, HD patients have higher pulse pressure (PP) values, a surrogate measure of vascular stiffness and an independent predictor of cardiovascular events and all-cause mortality at any mean arterial BP (MAP) level^25,26^. Thus, the big data for PP and MAP in HD patients can be computed via the following formulas from this dataset: SBP-DBP and DBP + (SBP-DBP)/3, respectively. Figure 6 shows the box plots for PP and MAP versus dialysis time with a 30-minute interval during HD treatment. These valuable data might be further applied to build a model to reduce the high PP level in the HD population.

## Supplementary information

Supplementary Information

## Data Availability

This implementation, built in the R statistical programming environment, was visualized by Shiny web application available at http://medi.dev.openlab.tw/data_analysis/. The scripts used for all data analyses, implemented in R-3.5.2, are available in the supplementary code information.
