# Supplementary material for: Dataset supporting blood pressure prediction for the management of chronic hemodialysis
Source: Sci Data. 2019 Dec 9;6:313. doi: 10.1038/s41597-019-0319-8 (PMC6901464; doi:10.1038/s41597-019-0319-8)
Supplement: Supplementary file 1 — Supplementary Information [file 41597_2019_319_MOESM1_ESM.doc]

Supplementary Code Information

**#The predictive code of BP prediction:**

data.d1<-read.csv('./Hemrec_D1.csv', header = TRUE)

data.vip<-read.csv('./VIP.csv', header = TRUE)

data.idp<-read.csv('./idp.csv', header = TRUE)

require(data.table)

setkey(data.d1, pid)

setkey(data.vip, pid)

setkey(data.idp, pid)

mergedata21<- data.vip[data.d1, nomatch=0]

mergedata4 <- mergedata21[data.idp, nomatch=0]

**#correlation plot code:** corrInput<- merge.datav4 colnames(corrInput)<-c('Measured

times','SBP','DBP','Dialysatetemperature','Dialysateconductivity','Ultrafiltration rate', 'Blood flow','Dialysistime','Gender','Age','Dialysisduration','Diabete','Body weight before HD','Body weight after HD','Dryweight','Body temperature')

M <- cor(corrInput, use = 'na.or.complete')

corrplot(M, method = 'circle')
